# Special Issue: “Enzymes and Enzyme Inhibitors—Applications in Medicine and Diagnosis 2.0”

**DOI:** 10.3390/ijms252413422

**Published:** 2024-12-14

**Authors:** Athina Geronikaki

**Affiliations:** School of Pharmacy, Aristotle University of Thessaloniki, 54124 Thessaloniki, Greece; geronik@pharm.auth.gr

## Introduction

The first paper in this Special Issue explores the synthesis, characterization, biological, and catalytic activities of new gold(I) and silver(I) complexes that are stabilized by caffeine derivatives and used as NHC ligands [1]. This subject is only tangentially related to the topic of this Special Issue but is included here due to the fact that silver(I) and gold(I) N-heterocyclic carbene (NHC) complexes have been attracting attention because of their catalytic activities. The anticancer activity of these complexes was evaluated against two breast cancer cell lines, resulting in a higher activity towards triple-negative MDA-MB-231, where they induced DNA damage due to the inhibition of human topoisomerase I (hTopoI) [2] and also regulated NF-κB localization [2] and TNF-α protein levels [3]. Furthermore, they reduced NO production in RAW 264.7 macrophages, as well as lowering iNOS and COX-2 expression levels, with a similar potency to indomethacin, an anti-inflammatory agent [4].

As Xanthine derivatives are known to possess interesting antioxidant and radical scavenging activities [5] that contribute to their biological properties, including their anticancer [6] and anti-inflammatory activities [4], the capability of the newly synthesized complexes to inhibit reactive oxygen species (ROS) production [7] was assessed using a cell-based assay employing a dihydro-2′,7′-dichlorofluorescein diacetate (H_2_DCF-DA) fluorescent probe. Some of the complexes, namely, AgL1OAc, AgL2OAc, and AuL1OAc, demonstrated remarkable antioxidant activity, leading to reductions in ROS levels of about 61, 59, and 54%, respectively. In addition, all of the synthesized complexes exerted an antioxidant capacity higher than those of caffeine and theophylline, which are used as reference compounds.

Finally, taking into account the fact that there are data showing the ability of caffeine and some derivatives to inhibit many strains of pathogenic bacteria [7], the newly synthesized complexes were investigated as potential antibacterials, as well. It was found that the Ag complexes were the most active.

The results obtained underline some of the possible applications of these complexes and pave the way for the expansion of their structural diversity, with the future aim of identifying useful and multi-action drug candidates.

The next paper in this Special Issue studies the role of α1-Antitrypsin (AAT) [8]. AAT, an acute-phase reactant not dissimilar to C-reactive protein (CRP), is a serine protease inhibitor that harbors tissue-protective and possibly immunomodulatory outcomes in patients with steroid-refractory graft-versus-host disease (GVHD) [9]. This disease represents a severe outcome of allogeneic hematopoietic stem cell transplantation (HSCT), a potentially curative intervention in hematological diseases. Taking this into account, the authors of this paper followed 53 patients listed for allogeneic HSCT (trial registration, NCT03188601) [10,11].

The authors then analyzed serum samples both before and after HSCT to measure AAT and CRP levels, along with intrinsic antiproteolytic activity. The ex vivo reaction to clinical-grade AAT was evaluated using circulating leukocytes from patients and a human epithelial cell line treated with patient sera in a gap closure assay. The results from the ex vivo studies indicate that circulating leukocytes exhibit a beneficial immune-regulated profile in response to AAT; furthermore, epithelial gap closure improves using AAT in sera from patients without GVHD, unlike those who have developed GVHD. Based on the serum collected prior to HSCT, non-relapse mortality could be accurately predicted by integrating three factors: AAT levels, CRP levels, and serum antiproteolytic activity. Overall, the outcomes of HSCT are significantly influenced by the antiproteolytic properties of circulating AAT, which supports the need for early AAT-increasing therapy in patients undergoing allogeneic HSCT.

The third paper in this Special Issue discusses the synthesis of 1H-chromeno [3,2-c]pyridine derivatives as MAO inhibitors with potential anticancer activity [12]. Chromone (i.e., 4*H*-chromen-4-one or 4H-1-benzopyran-4-one), a widely occurring oxaheterocyclic moiety from plants (e.g., flavonoids and xanthones), has gained importance as a ‘privileged’ scaffold in medicinal chemistry due to its drug likeness and differently ascertained pharmacological activities, such as its anti-inflammatory, antioxidant [13], neuroprotectant [14], anticancer [15], antimicrobial [15], and antiviral [16] activities. Based on their previous results on piperidine-fused chromone derivatives, which were evaluated against MAO-A and MAO-B enzymes, as well as against AChE, the authors synthesized about twenty molecules with a 1*H*-chromeno[3,2-c]pyridine scaffold. The aim was to prepare new chromeno[3,2-c]pyridine derivatives capable of expanding the toolbox of MAOs and AChE inhibitors for use as neuroprotectants in Alzheimer’s and/or Parkinson’s disease. Another goal was to evaluate the selected MAO B inhibitors against tumor growth inhibitory activity in three tumor cell lines, namely, breast (MCF-7), colon (HCT116), and cisplatin-resistant ovarian (SK-OV-3) tumor cells.

The compounds were tested as inhibitors of the human isoforms of MAO (A and B) and ChE (AChE and BChE), with each of them being tested against each enzyme. The MAO- B-selective inhibitor pargyline and the AChE-selective inhibitor galantamine were used as positive controls. According to the results obtained, only 1-(2-phenylethynyl), a derivative of 1,2-DHCP **3a**, achieved single-digit micromolar IC50 values against both ChEs, while all of the newly synthesized chromeno[3,2-c]pyridine derivatives exhibited very low activity as ChEs inhibitors. Some others appear to be good MAO-A inhibitors (IC_50_ in the range of 0.703–1.18 μM), being approximately tenfold selective over MAO-B, whereas the MAO-inhibitory activity of another, **c**, was affected by the substituents at N2 and C1.

Regarding antiproliferative activity evaluation, it was found that 10-indolyl THCP analogs **6a**–**c** were the most active among the tested compounds, with their selectivity against MCF-7 cell lines being IC50s (4.80 ÷ 6.82 μM), whereas compounds **2a**,**b** and **3b**,**c** exhibited lower activity in MCF-7 cell lines, being poorly active in SK-OV-3 cells (50 μM concentration).

In conclusion, 2-Cl/Br-substituted-8-methyl-2,3-dihydro-1*H*-chromeno[3,2-c]pyridine derivatives (**2a**,**b**) preferentially showed MAO-A inhibitory activity (IC_50_ 1 μM), whereas 2,6-dimethyl-1-(phenylethynyl)-1,2-dihydrobenzo[g]isoquinoline-5,10-dione derivatives (**3a**,**b**) were selective MAO-B inhibitors, with IC_50_ values of 0.51 and 0.63 μM, respectively. Furthermore, derivative 3a also exhibited inhibitory activity against both ChEs (IC_50_s of 7–8 μM) and 10-(1H-indol-3-yl)-bearing 2,3,4,10-THCP analogs, namely, compounds **6a**–**c**, achieving activity against three cell lines (MCF-7, HCT116, and SK-OV-3).

The fourth paper in this Special Issue discusses new hydrazide and hydrazine chelators that demonstrated rapid reactions towards iron cations with high sensitivity as a novel class of iron chelators and potential TET1 proteins (ten-eleven translocation methylcytosine dioxygenase 1) inhibitors [15,16,17]. Prompted by the results of their previous work on aromatic hydrazones that demonstrated promising inhibitory activity against the TET1 protein, and supported by docking studies, the authors decided to combine two pharmacophores and a fluorescent pyrrolo[3,2-b]pyrrole moiety with an appropriate metal chelating unit, which was then responsible for the inhibition of the TET1 protein, with the aim of preparing new TET1 protein inhibitors to characterize their Fe(II)-binding capacity, their cytotoxicity regarding cancer cell lines, and their level of expression of the TET1 protein. Furthermore, intracellular distributions and potential epigenetic effects were studied.

According to the obtained results, only one compound, **1**, showed inhibitory action, with an IC_50_ value of 1.33 μΜ, suggesting that the pyrrolo[3,2-b]pyrrole core could be useful in designing TET1 protein inhibitors.

Furthermore, the authors evaluated compounds **1**–**6** on the HF-P4, U-118 MG, H1299, BT-20, and Caov-3 cell lines using an MTT assay. The most cytotoxic appeared to be compounds **1 and 4**, with NH_2_ and one more OH group, respectively, indicating that a higher-polarity functionality can increase the cytotoxic effect.

The fifth paper in this Special Issue is an extensive review of the role of Protein Tyrosine Phosphatase 1B (PTP1B) in the pathogenesis of human diseases. This review provides an updated and comprehensive summary of the involvement of PTP1B expression-related disorders in the pathogenesis of human diseases [18].

The delineated characteristics of selected human diseases, as well as the involvement of PTP1B overexpression in the pathogenesis of these disorders, suggests that multidimensional mechanisms are accountable for the onset of each condition. Numerous elements of these related mechanisms overlap, producing synergic action or mediating signaling pathways via negative feedback. The authors highlight that while their association between diabetes type 2 (T2DM) and obesity seems rather clear, their connection with mental or neurodegenerative diseases is not apparent, and an improved understanding of these relationships may generate new opportunities in the treatment of the respective diseases. Although new inhibitors of PTP1B may seem like versatile treatments, caution is necessary in their routine pharmacotherapy use due to the uncertain role of the enzyme in the progression of certain cancer types. The authors point out that such decisions should always be based on reliable and thorough risk-to-benefit analyses.

Except for the above-mentioned diseases, the authors point out that the PTP1B enzyme is also involved in Alzheimer’s disease (AD), and its overexpression appears to be a critical factor in the onset of AD. Nevertheless, the role of PTP1B is not limited to these diseases; it is also involved in many others, such as major depressive disorders, fatty liver, and schizophrenia-like symptoms. Finally, PTP1B overexpression is also recognized as a therapeutic target in the treatment of potentially life-threatening stenosis of the aortic valve induced by calcific aortic valve disease.

Thus, PTP1B inhibitors, according to the authors and taking into account all the above-mentioned factors, can be useful in a wide range of applications and can also extend to areas currently lacking any therapeutic treatment [18,19,20,21,22,23,24,25].

Next, there is an extensive review on cyclic nucleotide phosphodiesterases 4 (PDE4) [26], a family of enzymes that precisely promote the hydrolysis and degradation of cAMP, interfering with the release or action of pro- and/or anti-inflammatory mediators, including tumor necrosis factor α (TNF-α), interleukin 10 (IL-10), and interleukin 12 (IL-12) [27], with several implications in the regulation of inflammatory responses. The authors refer to the history of the development of the first PDE4 inhibitors, and the main goal of this review is to aid in the selection of applicable medicinal chemistry steps in the development of PDE4 inhibitors (including dual agents). To achieve this, the authors analyze known PDF4 inhibitors of various chemical classes and discuss them in terms of their structural and pharmacophore features, as well as their binding properties, inhibitory activity, and, of course, their therapeutic applications. Despite the fact that, over the past 40 years, considerable advancements have been made in the discovery of PDE4 inhibitors, only a few of them have achieved approval for use as drugs. This, as the authors highlight, is due to the side effects observed during the development of several PDE4 inhibitors, mainly associated with a lack of specificity in their mechanisms of action. Another issue concerns the difficulties in generating PDE4 isoform-selective inhibitors because of a high degree of sequence and structural similarity among various subtypes, mainly those dealing with the catalytic site. The authors also mention that using non-selective isoforms offers the possibility of producing high efficacy in preclinical and clinical studies. A special report was written regarding dual inhibitors acting on several targets, opening a new avenue for new pharmacological applications. On the other hand, the simultaneous inhibition of PDE4 and other PDEs, as mentioned by the authors, led to synergistic effects, increasing both cAMP and cGMP levels, helping in the resolution of diseases affected by signaling pathways that involved these cyclic nucleotides.

Thus, with this review, the authors assist researchers in the selection of strategies, as well as structural requirements, aiding future research in molecular optimization strategies with the aim of identifying new candidates.

The next paper in this Special Issue explores the molecular mechanisms behind ACE2 signaling in pulmonary arterial hypertension (PAH) and discusses its potential as a therapeutic target. After introducing the readers to the fact that PAH is a devastating disorder characterized by an increase in pulmonary vascular resistance, which results in right-ventricular heart overload and failure, the authors discuss ACE2 signaling in PAH pathophysiology and molecular mechanisms [28,29,30]. The authors discuss in detail the role of the ACE/Ang II/AT1R signaling pathway and ACE 2, highlighting the fact that the damaging effects of the ACE/Ang II/AT1R signaling pathway in the cardiopulmonary system are compensated for by the vasoprotective ACE2/Ang-(1-7)/Mas 1 receptor signaling pathway. ACE2 is mainly expressed in the lungs, heart, and kidneys, being an ACE homologue, but differs from the latter in terms of substrate specificity and function. ACE2 acts as a carboxymonopeptidase, catalyzing the conversion of Ang I and II into the biologically active peptides Ang-(1-9) and Ang-(1-7), respectively. In turn, Ang-(1-7), the main product of ACE2, binds to the Mas 1 receptor, a G protein-coupled receptor. The authors summarize the ACE2 signaling-dependent molecular mechanisms involved in PAH pathophysiology [31,32,33,34].

Next, the authors highlight the clinical relevance of ACE2 signaling in PAH as a potential therapeutic target, giving some examples of preclinical studies that support this relevance.

Finally, the authors conclude that since ACE2 exerts a multitude of beneficial effects on the cardiopulmonary system, resulting in the prevention and reversal of PAH, genetically created approaches that upregulate ACE2 expression or activity and/or pharmacological ACE2 activators and recombinant human ACE2 may represent a novel therapeutic strategy in the management of PAH patients.

The last paper in this Special Issue is a review of recent developments in the inhibition of bacterial adhesion as a promising antivirulent strategy [35], keeping in mind the fact that chronic infections, often arising due to biofilm-forming pathogens, remain a major healthcare concern associated with high social and economic burdens. The authors mention in their paper that a valuable approach to counteracting antibiotic resistance consists of targeting bacterial virulence factors rather than killing pathogens. Virulence factors are bacterial molecules used by pathogens to colonize the host at the cellular level. These factors can be secretory, cytosolic, or membrane-associated. Thus, the authors discuss adhesion proteins as promising targets in preventing antibiotic resistance, showing the positive role of DsbA in several virulence factors for the relevant pathogens [36]. In Gram-negative bacteria, the main responsibility for adhesion falls on the pili, while in Gram-positive pathogens, this is the function of a class of proteins with structural motifs similar to those of pilin components, known to be microbial surface components that recognize adhesive matrix molecules (MSCRAMMs) [37]. Furthermore, the authors mention many strategies that have been suggested to contribute to a reduction in bacterial adhesion in order to hinder the infection process. A special report highlighted the role of SrtA [38]. In conclusion, in their review, the authors, taking into account the data from the literature used, mention that among the possible strategies proposed for the treatment of AMR infections caused by Gram-positive bacteria, SrtA inhibition can be considered to be one of the most valid approaches. Regarding the adhesion mechanism of Gram-negative bacteria, according to the authors, many targets can be evaluated, one of which is the development of molecules able to inhibit DSB oxidative protein-folding machinery.

As a summary of this review, the anti-adhesion agent can find numerous therapeutic applications, such as in prophylaxis in medical surgeries, as well as antibiotic adjuvants. Thus, there is an urgent need to develop new molecules as antibiotic adjuvants that can be used in combination with antibiotics, minimizing the effects of antibiotic resistance.
